# National Trends in the Prevalence of Chronic Kidney Disease Among Racial/Ethnic and Socioeconomic Status Groups, 1988-2016

**DOI:** 10.1001/jamanetworkopen.2020.7932

**Published:** 2020-07-16

**Authors:** Priya Vart, Neil R. Powe, Charles E. McCulloch, Rajiv Saran, Brenda W. Gillespie, Sharon Saydah, Deidra C. Crews

**Affiliations:** 1 Department for Health Evidence, Radboud Institute for Health Sciences, Radboud University Medical Center, Nijmegen, the Netherlands; 2 Zuckerberg San Francisco General Hospital and Trauma Center, Department of Medicine, University of California, San Francisco; 3 Department of Epidemiology and Biostatistics, School of Medicine, University of California, San Francisco; 4 Kidney Epidemiology and Cost Center, University of Michigan, Ann Arbor; 5 Division of Nephrology, Department of Medicine, University of Michigan, Ann Arbor; 6 Division of Diabetes Translation, Centers for Disease Control and Prevention, Atlanta, Georgia; 7 Division of Nephrology, Department of Medicine, Johns Hopkins University School of Medicine, Baltimore, Maryland; 8 Welch Center for Prevention, Epidemiology and Clinical Research, Johns Hopkins Medical Institutions, Baltimore, Maryland; 9 Johns Hopkins Center for Health Equity, Johns Hopkins Medical Institutions, Baltimore, Maryland

## Abstract

**Importance:**

The overall prevalence of chronic kidney disease (CKD) has stabilized in the United States in recent years. However, it is unclear whether all major sociodemographic groups experienced this trend.

**Objective:**

To examine trends in CKD prevalence across major sociodemographic groups as defined by race/ethnicity and socioeconomic status.

**Design, Setting, and Participants:**

This repeated cross-sectional study used data from the National Health and Nutrition Examination Surveys for 1988 to 1994 and every 2 years from 1999 to 2016 on individuals 20 years or older with information on race/ethnicity, socioeconomic status, and serum creatinine levels. Statistical analysis was conducted from May 1, 2017, to April 6, 2020.

**Exposures:**

Race/ethnicity and socioeconomic status.

**Main Outcomes and Measures:**

Prevalence of CKD was defined as an estimated glomerular filtration rate of 15 to 59 mL/min/1.73 m^2^.

**Results:**

A total of 54 554 participants (mean [SE] age, 46.2 [0.2] years; 51.7% female) were examined. The age-, sex- and race/ethnicity-adjusted overall prevalence of stage 3 and 4 CKD increased from 3.9% in 1988-1994 to 5.2% in 2003-2004 (difference, 1.3%; 95% CI, 0.9%-1.7%; *P* < .001 for change) and remained relatively stable thereafter at 5.1% in 2015-2016 (difference, −0.1%; 95% CI, −0.7% to 0.4%; *P* = .61 for change). The trend in adjusted CKD prevalence differed significantly by race/ethnicity (*P* = .009 for interaction). In non-Hispanic white and non-Hispanic black persons, CKD prevalence increased between 1988-1994 and 2003-2004 and remained stable thereafter. Among Mexican American persons, CKD prevalence was lower than in other racial/ethnic groups and remained stable between 1988-1994 and 2003-2004 but nearly doubled (difference, 2.1%; 95% CI, 0.9%-3.3%; *P* = .001 for change) between 2003-2004 and 2015-2016 to rates similar to those in other racial/ethnic groups. There were higher rates of CKD prevalence among groups with lower educational level and income (eg, 5.8% vs 4.3% and 4.3% vs 3.1% in low vs high education and income, respectively, in 1988-1994), but trends in CKD prevalence mirrored those for the overall population. The higher CKD prevalence among individuals with lower educational level and income remained largely consistent throughout the entire period. Results were similar in most subgroups when including albuminuria to define CKD.

**Conclusions and Relevance:**

The prevalence of CKD in the United States has stabilized overall in recent years but has increased among Mexican American persons. More important, gaps in CKD prevalence across racial/ethnic groups and levels of socioeconomic status largely persisted over 28 years. There is a need to identify and address causes of increasing CKD prevalence among Mexican American persons and a need to renew efforts to effectively mitigate persistent disparities in CKD prevalence.

## Key Points

**Question:**

What were the trends in prevalence of chronic kidney disease among all major sociodemographic groups in recent years?

**Findings:**

In this repeated cross-sectional study, gaps in chronic kidney disease prevalence across levels of socioeconomic status persisted over 28 years. Chronic kidney disease prevalence almost doubled among Mexican American persons between 2003-2004 and 2015-2016; other racial/ethnic and socioeconomic groups did not experience appreciable increases in the prevalence of chronic kidney disease.

**Meaning:**

There is a need to renew efforts to effectively mitigate persistent disparities in the prevalence of chronic kidney disease.

## Teaser

This repeated cross-sectional study uses data from the National Health and Nutrition Examination Surveys to examine trends in chronic kidney disease prevalence across major sociodemographic groups, as defined by race/ethnicity and socioeconomic status.

## Introduction

Chronic kidney disease (CKD) is associated with a disproportionately high risk of cardiovascular disease, progression to kidney failure requiring dialysis or transplantation, acute kidney injury, and death.^1,2,3,4,5,6^ Chronic kidney disease affects about 15% of the US adult population.^7^ Previous research has examined overall trends in CKD prevalence in the United States and has found no appreciable increase in CKD prevalence in recent years.^8^ Although this overall trend is encouraging, it is unclear whether CKD rates have stabilized across major sociodemographic groups for whom kidney failure rates have been high, such as those defined by race/ethnicity and socioeconomic status.

Moreover, Healthy People 2010 and 2020, the US national blueprint for public health goals, aimed to eliminate health disparities, including those associated with kidney disease, and achieve health equity. To examine progress toward this aim, the assessment of disparities in CKD prevalence is pivotal. Healthy People 2020 explicitly highlighted the importance of assessing health disparities by major sociodemographic factors in the US population in association with chronic conditions.^9^ In this study using a large, nationally representative sample of the US population, we aimed to investigate trends in CKD prevalence by race/ethnicity and socioeconomic status from 1988 to 2016 and investigate whether disparities persist.

## Methods

### Study Population

The National Health and Nutrition Examination Surveys (NHANES)^10^ are nationally representative surveys of the noninstitutionalized US population. In brief, NHANES III was conducted from 1988 to 1994; beginning in 1999, NHANES collected data continuously and released it in 2-year data cycles. In this study, we examined data from NHANES III (1988-1994) through NHANES 2015-2016. Participants 20 years or older with available information on their serum creatinine measurements, race/ethnicity, and socioeconomic status were included for analysis. Participants with CKD stage 5 (estimated glomerular filtration rate [eGFR] <15 mL/min/1.73 m^2^) were excluded owing to the small sample size and lack of information on participants’ dialysis history (eTable 1 in the Supplement). The research ethics boards of the National Center for Health Statistics approved NHANES. All participants provided written informed consent. Our report follows the Strengthening the Reporting of Observational Studies in Epidemiology (STROBE) reporting guideline for cross-sectional studies.^11^

### Sociodemographic Factors

Race/ethnicity and socioeconomic status (commonly assessed using educational level and poverty income ratio^12^) were considered as major sociodemographic variables of interest. Information on these 3 factors was self-reported. Race/ethnicity was categorized into 4 groups: non-Hispanic white, non-Hispanic black, Mexican American, and other. Educational level was categorized into 3 categories: less than high school (low), high school or equivalent (medium), and more than high school (high).^13^ Poverty income ratio (ratio of total household income divided by federal poverty income threshold corrected for household size and inflation) was categorized into less than 1.0 (low income), 1.0-3.9 (middle income), and 4.0 or more (high income).^14^

### Chronic Kidney Disease

The prevalence of stage 3 and 4 CKD (ie, eGFR 15-59 mL/min/1.73 m^2^)^15^ was the main outcome of interest. Glomerular filtration rate was estimated from serum creatinine using the Chronic Kidney Disease Epidemiology Collaboration equation.^16^ The calibrations recommended by the NHANES for serum creatinine measurements across time periods was used.^17^

### Statistical Analysis

Statistical analysis was conducted from May 1, 2017, to April 6, 2020. Following the NHANES’ data analysis protocol, our analyses included the recommended sample weights. Prevalence estimates were adjusted for age, sex, and race/ethnicity (except when analyzing race/ethnicity). In adjusted analyses, restricted cubic splines were used to flexibly model trends over time. To allow changes during the study period (1988-2016) and give the needed flexibility in shape, we used 3 predetermined knots at years 2000, 2004, and 2007.^8^ Temporal trends in CKD prevalence, with stratification by race/ethnicity, educational levels, and poverty income ratio, were examined with marginal-effect estimation.^18^ In this method, for example, the estimate of CKD prevalence across racial/ethnic groups over time was obtained by holding age and sex fixed at their observed values and varying race/ethnicity and survey year. Predicted probabilities (of having CKD for each year and for each person) obtained from fitted models were averaged to obtain the mean CKD risk for a given racial/ethnic group and year. The standard Wald tests from the sample survey logistic regression analysis in Stata, release 14 (StataCorp LLC) were used to calculate *P* values. To test for interactions of temporal trends across sociodemographic factors, we tested the combined interaction of the spline terms with race/ethnicity, educational level, and poverty income ratio. Values for graphing the spline fits and their SEs were derived using the “margins” command in Stata, release 14. All *P* values were from 2-sided tests, and results were deemed statistically significant at *P* < .05.

To assess the robustness of our results, a number of sensitivity analyses were performed. First, we examined trends in the prevalence of CKD redefined to include eGFR of 15 to 59 mL/min/1.73 m^2^ or a one-time urinary albumin to creatinine ratio of 30 mg/g or more. Second, we also assessed trends when using a more stringent cutoff to define CKD (ie, eGFR, 15-44 mL/min/1.73 m^2^). Third, trends in CKD prevalence were examined when additionally adjusting for diabetes and systolic blood pressure. Fourth, in cases in which a sociodemographic characteristic showed an interaction with time, we excluded the subgroup(s) that showed different trends and repeated the test for interaction.

## Results

 A total of 54 554 participants (mean [SE] age, 46.2 [0.2] years; 51.7% female) were examined. Characteristics of the study population are presented by survey period (Table 1).^16^ In later survey years, the mean (SE) age was higher (1988-1994, 44.4 [0.48] years; 2015-2016, 47.8 [0.57] years), the percentage of non-Hispanic white persons was lower (1988-1994, 77.7%; 2015-2016, 65.7%), and the proportion of Mexican American persons was higher (1988-1994, 4.8%; and 2015-2016, 8.5%). The proportion of the population with less than a high school education was also lower in later years (1988-1994, 23.7%; 2015-2016, 13.5%). Demographic characteristics of the study population by racial/ethnic groups are presented by survey period in eTable 2 in the Supplement. In later survey years, the mean age was higher, and there was a reduction in the percentage of those with less than a high school education across all racial/ethnic groups. Over the years, the trend in the proportion of those living below the poverty line was similar across racial/ethnic groups.

**Table 1.** Characteristics of Participants by Survey Years

Characteristic	Participants, No. (%)^a^									
	1988-1994 (n = 14 255)	1999-2000 (n = 3534)	2001-2002 (n = 4371)	2003-2004 (n = 4198)	2005-2006 (n = 4250)	2007-2008 (n = 5835)	2009-2010 (n = 5123)	2011-2012 (n = 4469)	2013-2014 (n = 4896)	2015-2016 (n = 4623)
Age, mean (SE), y	44.4 (0.48)	45.4 (0.38)	46.1 (0.51)	46.3 (0.51)	46.5 (0.73)	46.7 (0.43)	47.0 (0.51)	47.1 (0.87)	47.5 (0.38)	47.8 (0.57)
Female sex	7538 (51.8)	1862 (51.7)	2271 (51.7)	2161 (51.3)	2208 (52.0)	2452 (51.8)	2631 (51.6)	2260 (51.7)	2563 (51.5)	2398 (52.0)
Race/ethnicity										
Non-Hispanic white	6175 (77.7)	1629 (70.7)	2337 (73.5)	2241 (72.5)	2165 (72.8)	2362 (70.9)	2609 (70.5)	1745 (68.3)	2191 (67.4)	1593 (65.7)
Non-Hispanic black	3775 (10.0)	625 (9.8)	774 (9.8)	807 (10.9)	944 (11.0)	922 (10.0)	867 (10.4)	1109 (10.4)	956 (10.7)	930 (10.5)
Mexican American	3744 (4.8)	939 (7.1)	935 (6.9)	852 (7.8)	841 (7.7)	836 (8.3)	890 (8.1)	441 (7.5)	623 (8.6)	798 (8.5)
Other	561 (7.4)	341 (12.4)	325 (9.9)	298 (8.8)	300 (8.5)	715 (10.8)	757 (11.0)	1174 (13.7)	1126 (13.3)	1304 (15.2)
Educational level										
<High school	5626 (23.7)	1304 (23.0)	1297 (18.9)	1216 (17.9)	1152 (17.1)	1463 (20.1)	1413 (18.5)	976 (15.3)	1002 (14.6)	1037 (13.5)
High school	5318 (41.8)	795 (25.8)	1023 (25.2)	1042 (26.6)	1003 (24.6)	1176 (24.6)	1170 (22.8)	928 (19.7)	1107 (21.9)	1008 (20.7)
>High school	3311 (34.5)	1435 (51.2)	2051 (55.8)	1940 (55.5)	2095 (58.2)	2196 (55.3)	2540 (58.8)	2565 (65.0)	2788 (63.5)	2578 (65.8)
Income										
Low income	3304 (12.3)	719 (15.3)	749 (13.4)	784 (12.9)	754 (11.1)	1002 (14.0)	1154 (14.6)	1114 (17.3)	1104 (15.9)	1024 (14.2)
Middle income	8535 (60.4)	1902 (50.2)	2324 (48.9)	2316 (52.1)	2315 (51.6)	2593 (48.1)	2709 (49.3)	2209 (46.9)	2531 (49.5)	2508 (48.7)
High income	2416 (27.3)	913 (34.5)	1298 (37.7)	1098 (35.0)	1181 (37.2)	1240 (37.9)	1260 (36.0)	1146 (35.8)	1261 (34.7)	1091 (37.1)
eGFR, mean (SD), mL/min/1.73 m^2^^b^	99.3 (0.52)	94.3 (0.47)	94.2 (0.74)	94.1 (0.76)	93.9 (1.0)	95.7 (0.88)	94.6 (0.70)	94.9 (0.83)	93.3 (0.54)	95.6 (0.92)
ACR, mean (SD), mg/g	24.0 (1.4)	31.7 (6.1)	29.9 (2.8)	22.5 (2.4)	30.2 (3.0)	37.6 (4.4)	23.3 (2.9)	24.1 (2.1)	41.6 (4.8)	35.8 (3.3)

Abbreviations: ACR, albumin to creatinine ratio; eGFR, estimated glomerular filtration rate.

^a^ Percentages are weighted.

^b^ Estimated from serum creatinine using the Chronic Kidney Disease Epidemiology Collaboration equation.^16^

### Crude CKD Prevalence

The crude prevalence of stage 3 and 4 CKD (ie, eGFR 15-59 mL/min/1.73 m^2^) increased from 4.6% in 1988-1994 to 6.6% in 2003-2004 (difference, 2.0%; 95% CI, 1.2%-2.8%; *P* < .001 for change) (Table 2). The prevalence remained largely stable after 2003-2004, with a prevalence of 6.7% in 2015-2016 (difference, 0.1%; 95% CI, 0.9% to −1.1%; *P* = .83 for change).

**Table 2.** Crude Prevalence of Chronic Kidney Disease Among Adults 20 Years or Older Between 1988 and 2016^a^

Characteristic	1988-1994 (n = 14 255)	1999-2000 (n = 3534)	2001-2002 (n = 4371)	2003-2004 (n = 4198)	2005-2006 (n = 4250)	2007-2008 (n = 5835)	2009-2010 (n = 5123)	2011-2012 (n = 4469)	2013-2014 (n = 4896)	2015-2016 (n = 4623)
Overall, % (95% CI)	4.6 (4.0-5.1)	5.9 (5.5-6.4)	6.3 (5.8-6.8)	6.6 (6.1-7.1)	6.7 (6.2-7.2)	6.7 (6.3-7.1)	6.7 (6.3-7.1)	6.7 (6.3-7.1)	6.7 (6.1-7.3)	6.7 (6.0-7.4)
Race/ethnicity, % (95% CI)										
Non-Hispanic white	5.1 (4.4-5.8)	6.8 (6.3-7.3)	7.3 (6.7-7.8)	7.7 (7.0-8.3)	7.8 (7.3-8.4)	7.9 (7.4-8.3)	7.9 (7.4-8.4)	7.9 (7.3-8.5)	7.9 (7.2-8.9)	7.9 (7.0-8.9)
Non-Hispanic black	3.7 (3.2-4.2)	4.8 (4.3-5.3)	5.1 (4.4-5.7)	5.3 (4.6-6.1)	5.4 (4.7-6.1)	5.5 (4.9-6.0)	5.5 (5.0-6.0)	5.5 (5.0-6.1)	5.5 (4.8-6.2)	5.5 (4.7-6.4)
Mexican American	1.1 (0.7-1.4)	1.4 (1.1-1.7)	1.5 (1.1-1.9)	1.6 (1.1-2.0)	1.8 (1.3-2.2)	2.0 (1.6-2.4)	2.3 (1.8-2.8)	2.6 (2.1-3.2)	3.0 (2.2-3.8)	3.5 (2.4-4.6)
Other	2.9 (1.6-4.2)	3.6 (2.5-4.6)	3.7 (2.6-4.9)	3.9 (2.6-5.2)	3.9 (2.8-5.1)	3.9 (3.1-4.8)	3.9 (3.3-4.5)	3.9 (3.3-4.4)	3.8 (3.1-4.5)	3.8 (2.9-4.7)
Educational level, % (95% CI)										
<High school	9.1 (8.0-10.2)	10.4 (9.5-11.3)	10.7 (9.6-11.8)	10.9 (9.7-12.1)	10.7 (9.6-11.7)	10.3 (9.4-11.1)	9.8 (8.9-10.7)	9.4 (8.4-10.5)	9.0 (7.7-10.3)	8.6 (7.1-10.2)
High school	3.7 (3.1-4.3)	6.1 (5.5-6.6)	6.8 (6.1-7.5)	7.4 (6.6-8.3)	7.8 (7.0-8.5)	7.9 (7.2-8.5)	7.9 (7.4-8.5)	8.0 (7.4-8.6)	8.1 (7.3-8.9)	8.2 (7.1-9.2)
>High school	2.6 (2.0-3.2)	4.0 (3.5-4.5)	4.4 (3.8-5.0)	4.8 (4.1-5.4)	5.0 (4.4-5.7)	5.2 (4.7-5.7)	5.4 (4.9-5.8)	5.5 (5.0-6.1)	5.7 (5.0-6.4)	5.9 (4.9-6.8)
Income, % (95% CI)										
Low income	5.5 (4.4-6.6)	5.5 (4.8-6.2)	5.5 (4.7-6.3)	5.5 (4.7-6.4)	5.6 (4.8-6.3)	5.7 (5.1-6.3)	5.8 (5.2-6.5)	5.9 (5.1-6.8)	6.1 (4.9-7.2)	6.2 (4.7-7.7)
Middle income	5.1 (4.3-6.0)	7.5 (6.9-8.0)	8.1 (7.4-8.8)	8.6 (7.8-9.4)	8.6 (7.9-9.4)	8.4 (7.8-9.0)	8.2 (7.6-8.7)	7.9 (7.3-8.5)	7.7 (7.0-8.4)	7.5 (6.6-8.3)
High income	3.0 (2.1-3.8)	3.8 (3.2-4.4)	4.0 (3.4-4.7)	4.3 (3.6-5.0)	4.5 (3.9-5.2)	4.8 (4.2-5.3)	5.1 (4.5-5.6)	5.3 (4.7-6.0)	5.6 (4.7-6.5)	6.0 (4.7-7.2)

^a^ Chronic kidney disease defined as an estimated glomerular filtration rate of less than 60 mL/min/1.73 m^2^.

Crude CKD prevalence differed significantly by race/ethnicity (*P* = .04 for interaction). Among non-Hispanic white and non-Hispanic black persons, CKD prevalence increased between 1988-1994 (non-Hispanic white, 5.1%; non-Hispanic black, 3.7%) and 2003-2004 (non-Hispanic white, 7.7%; non-Hispanic black, 5.3%) but remained stable thereafter (Table 2). However, Mexican American persons experienced an appreciable increase in CKD prevalence from 2003-2004 (1.6%) to 2015-2016 (3.5%; difference, 1.9%; 95% CI, 0.6%-3.2%; *P* = .004 for change).

In 1988-1994, the crude CKD prevalence was highest among non-Hispanic white persons (5.1%) and lowest among Mexican American persons (1.1%; *P* < .001 for difference) (Table 2). In 2015-2016 as well, the crude CKD prevalence remained highest among non-Hispanic white persons (7.9%) and lowest among Mexican American persons (3.5%; *P* < .001 for difference).

The temporal trend in crude CKD prevalence also differed by levels of educational attainment and poverty income ratio (*P* < .001 for interaction and *P* = .002 for interaction, respectively). Across educational levels, a tendency toward decline in prevalence between 2003-2004 and 2015-2016 was observed in those with less than high school education (2003-2004, 10.9%; 2015-2016, 8.6%; difference, 2.3%; 95% CI, 0.04%-4.5%; *P* = .05 for change), and there was also a tendency toward an increase in CKD prevalence among those with more than high school education (2003-2004, 4.8%; 2015-2016, 5.9%; difference, 1.1%; 95% CI, −0.02% to 2.3%; *P* = .09 for change) (Table 2). Across levels of poverty income ratio, contrary to the overall trend, the CKD prevalence remained stable in low-income groups but increased throughout the entire period in those with high income (1988-1994, 3.0%; 2003-2004, 4.3%; difference, 1.3%; 95% CI, 0.3%-2.4%; *P* = .02 for change; 2003-2004, 4.3%; and 2015-2016, 6.0%; difference, 1.7%; 95% CI, 0.09%-3.3%; *P* = .04 for change).

In 1988-1994, individuals with less than high school education (9.1%) and those with low incomes (5.5%) had the highest CKD prevalence, while those with more than high school education (2.6%) and high income (3.0%) had the lowest CKD prevalence (*P* < .001 for difference in CKD prevalence between low and high educational levels and between low and high income). This trend was similar in 2015-2016.

### Adjusted CKD Prevalence

The age-, sex-, and race/ethnicity-adjusted overall prevalence of stage 3 and 4 CKD (ie, eGFR 15-59 mL/min/1.73 m^2^) increased from 3.9% in 1988-1994 to 5.2% in 2003-2004 (difference, 1.3%; 95% CI, 0.9%-1.7%; *P* < .001 for change) and remained relatively stable thereafter at 5.1% (difference, –0.1%; 95% CI, −0.7% to 0.4%) in 2015-2016 (*P* = .61 for change).

Chronic kidney disease prevalence trends differed significantly by race/ethnicity (*P* = .009 for interaction). Among non-Hispanic white persons and non-Hispanic black persons, CKD prevalence increased between 1988-1994 and 2003-2004 and remained stable thereafter. Among Mexican American persons, a greater increase in CKD prevalence was observed between 2003-2004 and 2015-2016 (difference, 2.1%; 95% CI, 0.9%-3.3%; *P* = .001 for change) (Figure, A). In 1988-1994, CKD prevalence was highest in non-Hispanic black persons (4.4%) and lowest in Mexican American persons (2.3%; *P* < .001 for difference). In 2015-2016, CKD prevalence was comparable among non-Hispanic black persons (5.7%) and Mexican American persons (5.1%; *P* = .55 for difference).

**Figure.**
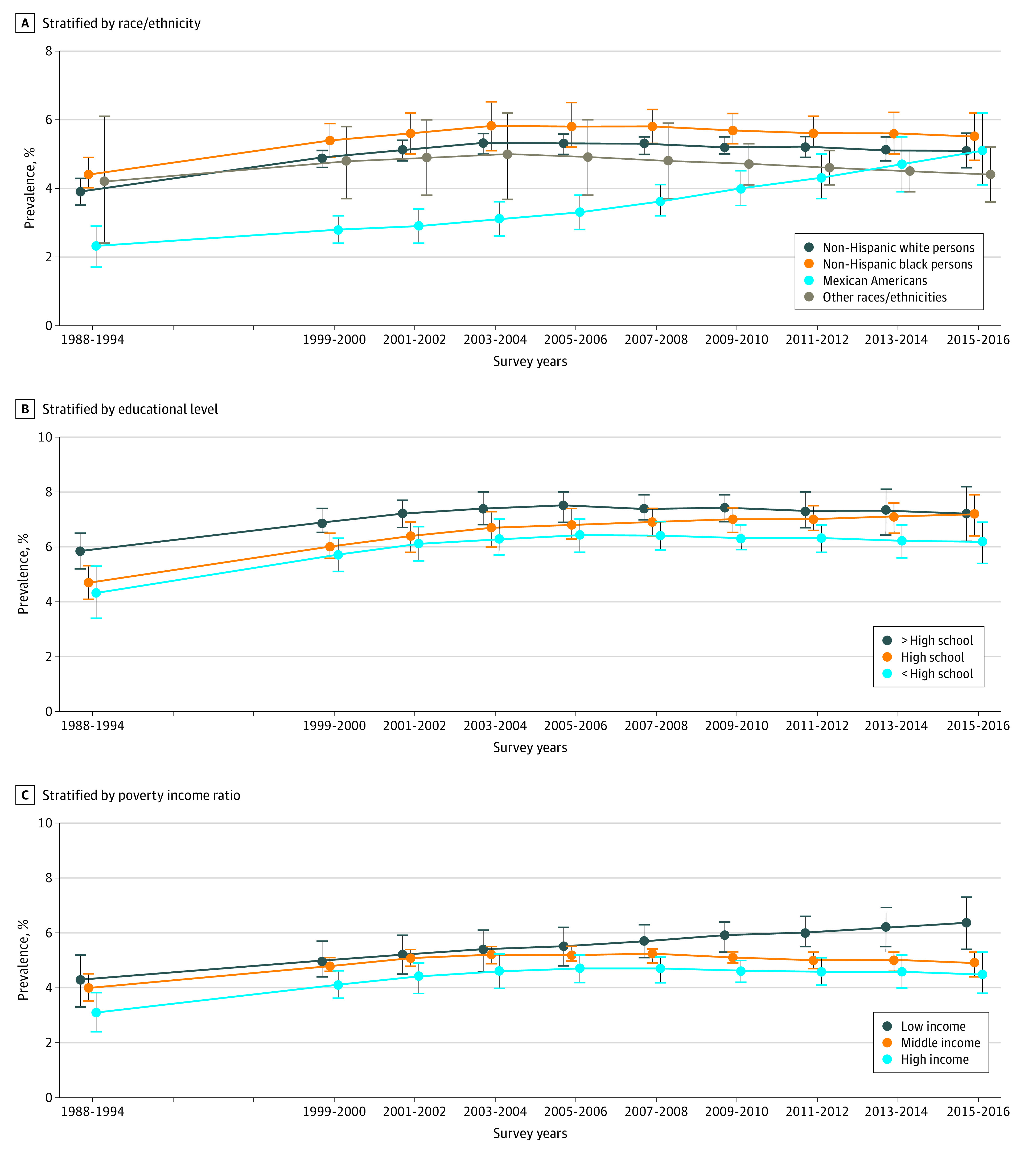
Age-, Sex- and Race/Ethnicity-Adjusted Prevalence of Stage 3 and 4 Chronic Kidney Disease in US Adults, 1988-2016 Prevalence was not adjusted for race/ethnicity when analyzing prevalence by race/ethnicity group. Other races/ethnicities included non–Mexican American Hispanic persons and Asian American persons. Dots indicate point estimates, and vertical lines indicate 95% CIs.

Trends in adjusted CKD prevalence among adults with less than high school education, high school education, and more than high school education followed the trend in CKD prevalence for the overall population (*P* = .38 for interaction), with similar results for low-income, middle-income, and high-income adults (*P* = .38 for interaction). The differences in CKD prevalence between those with less than high school education and those with more than high school education (Figure, B) and between low-income and high-income adults (Figure, C) remained largely persistent throughout the entire period. For instance, in 1988-1994 as well as in 2015-2016, CKD prevalence was highest among those with less than high school education and lowest in those with more than high school education (1999-2000, 5.8% vs 4.3%; *P* = .02 for difference; 2015-2016, 7.2% vs 6.2%; *P* = .09 for difference), with similar findings for low-income and high-income groups (1999-2000, 4.3% vs 3.1%; *P* = .07 for difference; 2015-2016, 6.4% vs 4.5%; *P* = .01 for difference).

### Adjusted CKD Prevalence With Expanded Definition

Using the expanded CKD definition (eGFR <60 mL/min/1.73 m^2^ and/or albuminuria ≥30 mg/g), overall CKD prevalence increased from 10.1% in 1988-1994 to 11.1% in 2003-2004 (difference, 0.9%; 95% CI, 0.1%-1.7%; *P* = .02 for difference) and remained stable thereafter, with a prevalence of 11.3% in 2015-2016 (difference, 0.2%; 95% CI, −0.7% to 1.2%) (Table 3). The temporal trend in CKD prevalence differed by racial/ethnic groups (*P* = .007 for interaction). Between 1988-1994 and 2015-2016, CKD prevalence increased most among Mexican American persons, although the most increase was observed between 1988-1994 (10.9%) and 2003-2004 (13.4%; difference, 2.4%; 95% CI, 0.6%-4.1%; *P* = .007 for difference). The temporal trend in CKD prevalence differed by education subgroups (*P* = .02 for interaction). Those with less than high school education experienced a decrease in CKD prevalence between 2003-2004 and 2015-2016 (17.4% vs 15.2%), and those with more than high school education experienced an increase in CKD prevalence between 2003-2004 and 2015-2016 (12.1% vs 13.4%). The temporal trend in CKD prevalence did not differ by income groups (*P* = .45 for interaction).

**Table 3.** Adjusted Prevalence of Chronic Kidney Disease With the Use of Expanded Definition Among Adults 20 Years or Older Between 1988 and 2014^a^

Characteristic	1988-1994 (n = 15 057)	1999-2000 (n = 3740)	2001-2002 (n = 4594)	2003-2004 (n = 4405)	2005-2006 (n = 4462)	2007-2008 (n = 5101)	2009-2010 (n = 5384)	2011-2012 (n = 4799)	2013-2014 (n = 5185)	2015-2016 (n = 4658)
Overall, % (95% CI)	10.1 (9.5-10.6)	10.7 (10.3-11.1)	10.8 (10.4-11.3)	11.1 (10.5-11.4)	11.0 (10.6-11.5)	11.1 (10.7-11.4)	11.1 (10.8-11.5)	11.1 (10.7-11.6)	11.2 (10.6-11.7)	11.3 (10.5-11.9)
Race/ethnicity, % (95% CI)										
Non-Hispanic white	9.2 (8.5-10.0)	9.8 (9.4-10.2)	9.9 (9.5-10.4)	10.1 (9.6-10.6)	10.2 (9.7-10.7)	10.3 (9.9-10.7)	10.4 (10.0-10.8)	10.5 (10.0-11.1)	10.7 (10.0-11.3)	10.8 (9.9-11.6)
Non-Hispanic black	14.1 (13.3-15.0)	14.0 (13.1-14.8)	13.9 (12.9-14.9)	13.9 (12.9-14.9)	13.9 (12.9-14.8)	13.8 (13.1-14.5)	13.8 (13.1-14.5)	13.7 (12.9-14.6)	13.7 (12.5-14.8)	13.6 (12.6-15.4)
Mexican American	10.9 (9.8-12.1)	12.6 (11.8-13.5)	13.0 (12.0-14.1)	13.4 (12.2-14.5)	13.5 (12.5-14.6)	13.6 (12.6-14.5)	13.6 (12.6-14.7)	13.7 (12.3-15.0)	13.7 (12.0-15.4)	13.7 (11.6-15.9)
Other	11.7 (8.8-14.6)	12.9 (11.4-14.4)	13.2 (11.5-14.9)	13.3 (11.4-15.3)	13.1 (11.3-14.8)	12.6 (11.3-13.9)	12.1 (11.1-13.1)	11.6 (10.8-12.5)	11.2 (10.2-12.2)	10.7 (9.5-12.0)
Educational level, % (95% CI)										
<High school	14.3 (12.9-15.8)	16.7 (15.7-17.5)	17.1 (16.1-18.1)	17.4 (16.4-18.5)	17.3 (16.4-18.3)	16.9 (16.0-17.7)	16.4 (15.7-17.2)	16.0 (15.1-16.9)	15.6 (14.4-16.7)	15.2 (13.9-16.5)
High school	12.4 (11.4-13.5)	13.5 (12.5-14.4)	13.7 (12.6-14.8)	14.0 (12.7-15.2)	14.2 (13.1-15.4)	14.5 (13.6-15.5)	14.8 (13.9-15.7)	15.1 (14.0-16.1)	15.4 (14.0-16.7)	15.7 (14.0-17.4)
>High school	10.9 (9.7-12.0)	11.7 (11.0-12.4)	11.9 (11.1-12.6)	12.1 (11.2-12.9)	12.3 (11.5-13.0)	12.5 (11.9-13.1)	12.7 (12.1-13.3)	12.9 (12.2-13.6)	13.2 (12.3-14.1)	13.4 (12.2-14.5)
Income, % (95% CI)										
Low income	13.4 (11.9-15.1)	14.2 (12.9-15.5)	14.4 (12.9-15.9)	14.5 (13.0-16.1)	14.6 (13.2-16.0)	14.5 (13.4-15.6)	14.5 (13.6-15.4)	14.4 (13.5-15.3)	14.4 (13.2-15.5)	14.3 (12.8-15.8)
Middle income	9.9 (9.2-10.7)	11.1 (10.6-11.6)	11.4 (10.8-12.0)	11.6 (11.0-12.3)	11.7 (11.1-12.3)	11.7 (11.2-12.2)	11.6 (11.1-12.1)	11.5 (11.0-12.1)	11.5 (10.8-12.2)	11.4 (10.6-12.3)
High income	8.0 (7.1-8.9)	8.2 (7.6-8.7)	8.2 (7.5-8.8)	8.3 (7.6-9.0)	8.4 (7.8-9.0)	8.6 (8.1-9.1)	8.8 (8.2-9.4)	9.0 (8.2-9.8)	9.2 (8.1-10.3)	9.4 (8.0-10.8)

^a^ Expanded definition of chronic kidney disease: estimated glomerular filtration rate of less than 60 mL/min/1.73 m^2^ and/or albumin to creatinine ratio of 30 mg/g or greater.

### Additional Sensitivity Analyses

When using a more rigorous CKD definition (ie, eGFR 15-45 mL/min/1.73 m^2^), results were similar to the results obtained when using our primary definition (eTable 3 in the Supplement). When additionally adjusting for diabetes and systolic blood pressure, CKD prevalence differed significantly by race/ethnicity (*P* = .002 for interaction) (eTable 4 in the Supplement). During the study period, the prevalence of CKD increased most among Mexican American persons (2003-2004, 3.4%; 2015-2016, 5.2%; *P* = .01 for change). Among non-Hispanic white persons, the CKD prevalence was 5.8% in 2003-2004 and was 5.2% in 2015-2016 (*P* = .19 for change), and among non-Hispanic black persons, the CKD prevalence was 6.8% in 2003-2004 and was 5.4% in 2015-2016 (*P* = .05 for change). Temporal trends in CKD prevalence did not differ by educational and poverty income ratio groups (*P* = .37 and *P* = .32, respectively, for interaction). After excluding Mexican American persons from the analysis, the interaction between race and time was not statistically significant (*P* = .90 for interaction).

## Discussion

In this national study, we examined temporal trends in CKD prevalence for major US sociodemographic groups, defined by race/ethnicity, educational level, and income. Results suggest that CKD prevalence in the United States has stabilized in recent years, both overall and for all racial/ethnic strata, with the exception of Mexican American persons, who continued to experience increases in CKD prevalence during this period. Moreover, gaps in CKD prevalence across educational and income levels appeared to have persisted in recent decades.

Our study extends an earlier study documenting temporal trends in overall CKD prevalence in the United States by noting that particular subgroups of persons are vulnerable. Although the previous study by Murphy et al^8^ included analyses by racial/ethnic groups and found no statistically significant difference in CKD prevalence, Mexican American persons were not examined separately from racial/ethnic groups categorized as “other.” In addition, our study found no appreciable narrowing of the gap in CKD prevalence across levels of income and educational attainment between 1988 and 2016.

The increasing CKD prevalence observed among Mexican American persons is consistent with the recently observed worsening health status among Hispanic individuals compared with non-Hispanic white persons, which is in contrast to what some have described as the “Hispanic paradox” (ie, Hispanic individuals having health outcomes that are better than or comparable with those of non-Hispanic white persons despite living in less favorable conditions).^19^ Observed results for Mexican American persons are unlikely to be due to racial/ethnic differences in the burden of diabetes and hypertension, as there were statistically significant differences in CKD prevalence even in analyses adjusting for these conditions. Migration patterns (immigration and emigration) of Mexican American persons may be a factor associated with the results. With the increasing racial/ethnic diversity of the US population, there is a need to identify and address the underlying causes of this trend.

Our observation of higher CKD prevalence at any time point among persons with low educational or low income levels updates the findings of prior work.^20,21^ However, the observation demonstrates consistent gaps in CKD prevalence across these groups over nearly 3 decades. Although the aim of this study was not to elucidate causes, it is conceivable that consistent inequality in CKD prevalence could be in part owing to health-promoting interventions not targeting specific at-risk groups or not being specifically designed to be capable of eliminating or reducing disparities.^22,23,24,25^ For instance, clustering of interventions (eg, bicycle-supporting infrastructure promoting physical activity) in neighborhoods whose residents have high median incomes and/or high employment rates may worsen health disparities experienced by residents of less advantaged communities.^26^ Our study has several research and public health implications. Our findings highlight the need to focus on the increasing CKD prevalence in Mexican American individuals. There may also be a need for enhanced efforts aimed at narrowing racial/ethnic and socioeconomic disparities in CKD prevalence. Some strategies have proven successful in decreasing rates of CKD among racial/ethnic minority populations (eg, the Indian Health Service program for lowering rates of end-stage kidney disease among persons with diabetes)^27,28^ and individuals with low socioeconomic status (eg, interventions targeting education and early childhood)^29^ and should be explored for their effectiveness and broader implementation. Although the overall CKD prevalence has stabilized in recent years, CKD awareness continues to remain very low and is even lower in some age and racial/ethnic groups than others.^30^ Future studies should investigate secular trends in CKD awareness by sociodemographic groups and identify factors associated with low CKD awareness. Moreover, identifying underlying risk factors explaining the observed trend could be relevant in terms of clinical outcomes and policy. Future studies should investigate temporal trends in CKD risk factors across sociodemographic subgroups.

### Strengths and Limitations

Our study had several strengths. The data are nationally representative, which allows them to be generalizable to the entire US noninstitutionalized adult population. The data were collected using standardized procedures on a wide range of factors, including demographic characteristics and comorbid conditions, and in a uniform manner throughout the study years. In addition, we performed NHANES-recommended calibration of serum creatinine measurements for a comparable assessment of kidney function across survey years.^16^

Our study also has some limitations, including a lack of sufficient data on other racial/ethnic groups in the United States, including American Indian, Asian, and Pacific Islander individuals, as well as other subpopulations of Hispanic persons. Thus, we were not able to examine these subgroups. In addition, because there was only 1 measurement of serum creatinine or albuminuria in NHANES, as well as the use of estimated rather than measured glomerular filtration rate, there was a possibility of misclassification of CKD status. However, misclassification is likely to be nondifferential across survey years, making it unlikely to have resulted in a biased assessment of temporal trends. Also, evidence suggests that there is no appreciable bias in CKD staging based on only the first measurement compared with more than 1 measurement.^31^ Our primary conclusion is based on CKD prevalence defined by eGFR alone, which also meant that CKD prevalence was lower than 10%, which is the prevalence when CKD is defined using both eGFR and albuminuria. This finding was owing to the lack of a standardized assay and inaccuracy of assays for albuminuria, which raises the potential for bias, especially when assays are performed years apart and the aim is to assess temporal trends. Because NHANES does not include data on duration and history of CKD, it was not possible to investigate any difference in sociodemographic factors between subgroups of patients with CKD of various duration and history.

## Conclusions

The prevalence of CKD in the United States has stabilized in recent years among most racial/ethnic groups but not among Mexican American persons. More important, gaps in CKD prevalence by racial/ethnic groups and socioeconomic status were largely persistent. To achieve health equity, renewed efforts are needed to effectively mitigate persistent disparities in CKD prevalence.

## Notes

Supplement. **eTable 1.** Number of Participants Excluded by Race **eTable 2.** Demographic Characteristics by Racial/Ethnic Groups Across Survey Years **eTable 3.** Adjusted Prevalence of Chronic Kidney Disease (eGFR<45 ml/min/1.73m^2^) Among Adults Aged 20 Years or More Between 1988 and 2014, by Race/Ethnicity, and Levels of Education and Poverty Income Ratio **eTable 4.** Adjusted Prevalence of Chronic Kidney Disease (eGFR<60 ml/min/1.73m^2^) Among Adults Aged 20 Years or More Between 1988 and 2014, by Race/Ethnicity, and Levels of Education and Poverty Income Ratio (Adjusted for Age, Sex, Race, Diabetes, Systolic Blood Pressure)
