# Error in Figure

**DOI:** 10.1001/jamanetworkopen.2020.19157

**Published:** 2020-08-17

In the Original Investigation titled “National Trends in the Prevalence of Chronic Kidney Disease Among Racial/Ethnic and Socioeconomic Status Groups, 1988-2016,”^1^ published on July 16, 2020, the key to panel B of the Figure was incorrectly labeled. The dark blue line indicates an educational level of less than high school (and should be labeled “<High school”), and the light blue line indicates an educational level of more than high school (and should be labeled “>High school”). This article has been corrected.
