# Supplementary material for: National Trends in the Prevalence of Chronic Kidney Disease Among Racial/Ethnic and Socioeconomic Status Groups, 1988-2016
Source: JAMA Netw Open. 2020 Jul 16;3(7):e207932. doi: 10.1001/jamanetworkopen.2020.7932 (PMC7366187; doi:10.1001/jamanetworkopen.2020.7932)
Supplement: Supplement. — eTable 1. Number of Participants Excluded by Race eTable 2. Demographic Characteristics by Racial/Ethnic Groups Across Survey Years eTable 3. Adjusted Prevalence of Chronic Kidney Disease (eGFR<45 ml/min/1.73m2) Among Adults Aged 20 Years or More Between 1988 and 2014, by Race/Ethnicity, and Levels of Education and Poverty Income Ratio eTable 4. Adjusted Prevalence of Chronic Kidney Disease (eGFR<60 ml/min/1.73m2) Among Adults Aged 20 Years or More Between 1988 and 2014, by Race/Ethnicity, and Levels of Education and Poverty Income Ratio (Adjusted for Age, Sex, Race, Diabetes, Systolic Blood Pressure) [file jamanetwopen-3-e207932-s001.pdf]

Supplementary Online Content

Vart P, Powe NR, McCulloch CE, et al; Centers for Disease Control and Prevention Chronic Kidney Disease Surveillance Team. National trends in the prevalence of chronic kidney disease among racial/ethnic and socioeconomic status groups, 1988-2016. *JAMA Netw Open.* 2020;3(7):e207932. doi:10.1001/jamanetworkopen.2020.7932

eTable 1. Number of Participants Excluded by Race
eTable 2. Demographic Characteristics by Racial/Ethnic Groups Across Survey Years
eTable 3. Adjusted Prevalence of Chronic Kidney Disease (eGFR<45 ml/min/1.73m^2) Among Adults Aged 20 Years or More Between 1988 and 2014, by Race/Ethnicity, and Levels of Education and Poverty Income Ratio
eTable 4. Adjusted Prevalence of Chronic Kidney Disease (eGFR<60 ml/min/1.73m^2) Among Adults Aged 20 Years or More Between 1988 and 2014, by Race/Ethnicity, and Levels of Education and Poverty Income Ratio (Adjusted for Age, Sex, Race, Diabetes, Systolic Blood Pressure)

This supplementary material has been provided by the authors to give readers additional information about their work.

© 2020 Vart P et al. *JAMA Network Open.*

eTable 1. Number of Participants Excluded by Race

	NH-White	NH-blacks	Mexican-Americans	Others
**Total population**	**42,765**	**27,015**	**26,388**	**15,944**
**Total excluded**	**17,718**	**15,306**	**15,489**	**9,045**
**eGFR missing***	**3,663**	**2,297**	**1,419**	**1,022**
**ACR missing***	**2,654**	**1,111**	**935**	**563**
**eGFR<15***	**40**	**84**	**34**	**14**

***among those 20 years and older**

© 2020 Vart P et al. *JAMA Network Open.*

eTable 2. Demographic Characteristics by Racial/Ethnic Groups Across Survey Years

**Characteristics**		**1988-1994 (n=15,082)**	**1999-2000 (3,760)**	**2001-2002 (4,608)**	**2003-2004 (4,412)**	**2005-2006 (4,473)**	**2007-2008 (5,110)**	**2009-2010 (5,402)**	**2011-2012 (4,817)**	**2013-2014 (5,205)**	**2015-2016 (4,668)**
**Age**											
-Non-Hispanic whites		45.6 ± 0.62	47.0 ± 0.50	47.8 ± 0.56	48.0 ± 0.59	48.2 ± 0.84	48.4 ± 0.47	48.9 ± 0.57	49.2 ± 0.95	49.7 ± 0.46	49.8 ± 0.73
-Non-Hispanic blacks		41.3 ± 0.41	42.8 ± 0.40	42.5 ± 0.61	43.2 ± 0.43	43.9 ± 0.91	44.5 ± 0.74	44.2 ± 1.07	44.0 ± 1.07	45.0 ± 0.94	44.8 ± 0.69
-Mexican-Americans		37.4 ± 0.41	38.9 ± 0.97	37.2 ± 0.87	38.6 ± 1.52	39.0 ± 0.78	39.5 ± 0.88	40.8 ± 0.91	39.3 ± 0.61	41.1 ± 1.01	41.9 ± 1.24
-Others		40.8 ± 0.90	41.8 ± 0.82	43.0 ± 1.65	43.1 ± 0.93	42.5 ± 0.99	43.4 ± 1.10	41.7 ± 0.81	43.2 ± 0.90	42.6 ± 0.70	44.2 ± 1.03
**Sex (female)**											
-Non-Hispanic whites		51.6	51.2	51.7	51.3	51.7	52.1	51.4	51.3	51.0	51.8
-Non-Hispanic blacks		55.2	55.7	54.1	55.3	54.7	55.8	56.1	57.5	55.6	55.7
-Mexican-Americans		47.8	49.0	45.2	47.5	47.1	46.5	47.3	46.9	47.5	49.7
-Others		52.4	52.6	54.3	49.7	55.6	50.1	52.1	51.7	53.4	51.3
**Education (<HS)**											
-Non-Hispanic whites		19.5	15.3	13.5	11.7	11.6	14.6	13.0	10.2	9.7	7.7
-Non-Hispanic blacks		30.6	37.9	31.9	28.9	23.8	27.5	24.3	17.6	20.2	15.6
-Mexican-Americans		56.5	56.2	53.2	50.4	54.6	48.4	51.8	45.8	43.7	43.8
-Others		36.5	36.4	22.8	26.6	22.1	27.3	23.6	22.6	16.0	19.8
**Income (poor)**											
-Non-Hispanic whites		7.9	10.0	8.9	9.1	7.2	10.2	9.5	12.0	11.0	8.3
-Non-Hispanic blacks		28.9	25.7	25.7	24.5	19.5	20.9	25.1	30.2	29.6	26.0
-Mexican-Americans		34.3	29.2	25.8	28.5	33.4	30.6	34.8	33.9	30.5	32.7
-Others		21.9	28.9	26.6	16.0	13.4	20.5	23.1	24.7	20.3	21.2

© 2020 Vart P et al. *JAMA Network Open.*

eTable 3. Adjusted Prevalence of Chronic Kidney Disease (eGFR<45 ml/min/$1.73m^2$) Among Adults Aged 20 Years or More Between 1988 and 2014, by Race/Ethnicity, and Levels of Education and Poverty Income Ratio

**Characteristics**		**1988-1994 (n=15,082)**	**1999-2000 (3,760)**	**2001-2002 (4,608)**	**2003-2004 (4,412)**	**2005-2006 (4,473)**	**2007-2008 (5,110)**	**2009-2010 (5,402)**	**2011-2012 (4,817)**	**2013-2014 (5,205)**	**2015-2016 (4,668)**
**Overall, %**		1.1 (1.0-1.1)	1.4 (1.3-1.5)	1.5 (1.3-1.6)	1.5 (1.4-1.7)	1.6 (1.4-1.7)	1.6 (1.5-1.7)	1.7 (1.5-1.8)	1.7 (1.5-1.9)	1.8 (1.5-2.0)	1.8 (1.5-2.1)
**Race, %**											
-Non-Hispanic whites		1.1 (0.9-1.3)	1.4 (1.2-1.5)	1.4 (1.3-1.6)	1.5 (1.3-1.7)	1.5 (1.4-1.7)	1.6 (1.4-1.7)	1.6 (1.5-1.7)	1.6 (1.4-1.8)	1.7 (1.4-1.9)	1.7 (1.4-2.0)
-Non-Hispanic blacks		1.7 (1.4-2.1)	2.0 (1.6-2.3)	2.0 (1.6-2.5)	2.1 (1.6-2.6)	2.2 (1.7-2.6)	2.2 (1.9-2.6)	2.3 (2.0-2.7)	2.4 (2.0-2.8)	2.5 (1.9-3.1)	2.6 (1.8-3.4)
-Mexican-Americans		1.0 (0.6-1.4)	0.9 (0.7-1.1)	0.9 (0.6-1.1)	0.9 (0.6-1.2)	1.1 (0.8-1.4)	1.4 (1.1-1.7)	1.7 (1.4-2.1)	2.2 (1.7-2.7)	2.7 (2.0-3.5)	3.4 (2.4-4.5)
-Others		1.2 (0.3-2.0)	1.4 (0.8-2.1)	1.5 (0.8-2.2)	1.6 (0.8-2.3)	1.6 (0.9-2.2)	1.6 (1.1-2.1)	1.6 (1.1-2.0)	1.6 (1.0-2.1)	1.5 (0.8-2.3)	1.5 (0.6-2.5)
**Education, %**											
-<High school		1.5 (1.2-1.8)	1.8 (1.6-2.1)	1.9 (1.6-2.2)	2.0 (1.6-2.4)	2.2 (1.9-2.6)	2.5 (2.2-2.8)	2.8 (2.4-3.2)	3.2 (2.6-3.7)	3.5 (2.7-4.4)	3.9 (2.7-5.1)
-High school		1.5 (1.2-1.8)	2.1 (1.8-2.4)	2.2 (1.8-2.6)	2.3 (1.9-2.8)	2.4 (2.1-2.8)	2.5 (2.2-2.7)	2.5 (2.2-2.8)	2.5 (2.1-2.9)	2.6 (2.0-3.1)	2.6 (1.9-3.3)
->High school		1.4 (0.8-1.9)	1.6 (1.3-1.8)	1.6 (1.3-1.9)	1.6 (1.3-2.0)	1.7 (1.4-1.9)	1.7 (1.4-1.9)	1.7 (1.5-1.9)	1.7 (1.4-1.9)	1.7 (1.4-1.9)	1.7 (1.3-2.0)
**Income, %**											
-Poor		1.4 (0.9-1.8)	1.5 (1.1-1.9)	1.5 (1.0-2.1)	1.6 (1.1-2.2)	1.8 (1.3-2.3)	2.0 (1.6-2.4)	2.2 (1.8-2.7)	2.5 (1.9-3.1)	2.8 (1.9-3.7)	3.2 (1.8-4.5)
-Middle income		1.2 (1.0-1.4)	1.4 (1.3-1.6)	1.5 (1.3-1.7)	1.6 (1.4-1.8)	1.6 (1.4-1.8)	1.7 (1.5-1.9)	1.8 (1.6-1.9)	1.8 (1.6-2.1)	1.9 (1.5-2.2)	1.9 (1.5-2.4)
-High income		0.7 (0.4-1.0)	1.1 (0.8-1.3)	1.2 (0.9-1.4)	1.2 (0.9-1.5)	1.2 (1.0-1.4)	1.1 (0.9-1.3)	1.0 (0.9-1.2)	1.0 (0.8-1.2)	0.9 (0.7-1.2)	0.8 (0.5-1.1)

© 2020 Vart P et al. *JAMA Network Open.*

eTable 4. Adjusted Prevalence of Chronic Kidney Disease (eGFR<60 ml/min/1.73m^2) Among Adults Aged 20 Years or More Between 1988 and 2014, by Race/Ethnicity, and Levels of Education and Poverty Income Ratio (Adjusted for Age, Sex, Race, Diabetes, Systolic Blood Pressure)

**Characteristics**		**1988-1994 (n=10,407)**	**1999-2000 (2,746)**	**2001-2002 (3,409)**	**2003-2004 (2,805)**	**2005-2006 (3,205)**	**2007-2008 (4,353)**	**2009-2010 (4,715)**	**2011-2012 (4,161)**	**2013-2014 (4,597)**	**2015-2016 (4,437)**
**Overall, %**		4.2 (3.7-4.7)	5.3 (5.0-5.7)	5.6 (5.2-6.0)	5.8 (5.4-6.2)	5.8 (5.4-6.2)	5.7 (5.4-5.9)	5.5 (5.3-5.8)	5.4 (5.1-5.7)	5.3 (4.9-5.7)	5.2 (4.6-5.7)
**Race**, %											
-Non-Hispanic whites		4.2 (3.7-4.7)	5.3 (4.9-5.7)	5.6 (5.2-6.0)	5.8 (5.3-6.2)	5.8 (5.4-6.2)	5.7 (5.4-6.0)	5.6 (5.3-5.9)	5.4 (5.1-5.8)	5.3 (4.9-5.8)	5.2 (4.6-5.8)
-Non-Hispanic blacks		4.9 (4.2-5.6)	6.2 (5.6-6.9)	6.6 (5.8-7.4)	6.8 (5.9-7.6)	6.7 (5.9-7.5)	6.4 (5.8-7.0)	6.2 (5.7-6.7)	5.9 (5.4-6.4)	5.7 (5.0-6.3)	5.4 (4.6-6.2)
-Mexican-Americans		2.2 (1.5-2.9)	2.9 (2.4-3.4)	3.1 (2.5-3.7)	3.4 (2.7-4.0)	3.6 (3.0-4.3)	3.9 (3.3-4.5)	4.2 (3.6-4.8)	4.5 (3.8-5.2)	4.9 (4.0-5.8)	5.2 (4.1-6.4)
-Others		5.2 (2.5-7.8)	6.0 (4.4-7.5)	6.2 (4.5-7.8)	6.2 (4.5-7.9)	6.0 (4.5-7.5)	5.6 (4.6-6.7)	5.3 (4.5-6.0)	4.9 (4.4-5.5)	4.6 (4.0-5.3)	4.3 (4.5-5.2)
**Education, %**											
-<High school		5.7 (4.8-6.5)	6.8 (6.2-7.5)	7.1 (6.4-7.8)	7.3 (6.6-8.1)	7.3 (6.7-8.0)	7.2 (6.6-7.7)	7.0 (6.4-7.6)	6.8 (6.1-7.6)	6.7 (5.7-7.6)	6.5 (5.4-7.6)
-High school		4.6 (3.9-5.2)	5.8 (5.2-6.3)	6.1 (5.4-6.7)	6.3 (5.6-7.1)	6.5 (5.8-7.1)	6.5 (6.0-7.0)	6.5 (6.1-7.0)	6.6 (6.1-7.0)	6.6 (6.0-7.2)	6.6 (5.8-7.4)
->High school		4.1 (3.1-5.2)	5.8 (5.1-6.5)	6.3 (5.5-7.0)	6.6 (5.8-7.4)	6.6 (5.9-7.3)	6.4 (5.9-7.0)	6.2 (5.7-6.7)	6.0 (5.5-6.6)	5.8 (5.1-6.5)	5.6 (4.8-6.5)
**Income, %**											
-Poor		4.4 (3.0-5.8)	5.4 (4.5-6.3)	5.7 (4.7-6.7)	5.9 (4.8-7.0)	6.0 (5.0-7.0)	6.1 (5.3-6.9)	6.2 (5.5-6.8)	6.3 (5.6-6.9)	6.3 (5.5-7.2)	6.4 (5.3-7.5)
-Middle income		4.5 (3.8-5.0)	5.4 (4.9-5.8)	5.6 (5.2-6.0)	5.8 (5.3-6.2)	5.7 (5.3-6.1)	5.6 (5.3-5.9)	5.4 (5.1-5.7)	5.2 (4.9-5.6)	5.1 (4.6-5.5)	4.9 (4.3-5.5)
-High income		3.1 (2.2-4.1)	4.6 (4.0-5.3)	5.0 (4.3-5.7)	5.3 (4.5-6.1)	5.4 (4.7-6.1)	5.3 (4.7-5.8)	5.1 (4.7-5.6)	5.0 (4.5-5.5)	4.9 (4.2-5.6)	4.7 (3.9-5.6)

© 2020 Vart P et al. *JAMA Network Open.*
